# Advances in the Understanding of Two-Pore Domain TASK Potassium Channels and Their Potential as Therapeutic Targets

**DOI:** 10.3390/molecules27238296

**Published:** 2022-11-28

**Authors:** Xueming Fan, Yongzhi Lu, Guizhi Du, Jin Liu

**Affiliations:** 1 Laboratory of Anesthesia and Critical Care Medicine, National-Local Joint Engineering Research Center of Translational Medicine of Anesthesiology, Department of Anesthesiology, West China Hospital, Sichuan University, Chengdu 610041, China; 2 Department of Anesthesiology, Guizhou Provincial People’s Hospital, Guiyang 550002, China; 3 Guangdong Provincial Key Laboratory of Biocomputing, Guangzhou Institute of Biomedicine and Health, Chinese Academy of Sciences, Guangzhou 510700, China

**Keywords:** TASK channels, biophysical properties, gating profiles, biological roles, targeted compounds

## Abstract

TWIK-related acid-sensitive K^+^ (TASK) channels, including TASK-1, TASK-3, and TASK-5, are important members of the two-pore domain potassium (K_2P_) channel family. TASK-5 is not functionally expressed in the recombinant system. TASK channels are very sensitive to changes in extracellular pH and are active during all membrane potential periods. They are similar to other K_2P_ channels in that they can create and use background-leaked potassium currents to stabilize resting membrane conductance and repolarize the action potential of excitable cells. TASK channels are expressed in both the nervous system and peripheral tissues, including excitable and non-excitable cells, and are widely engaged in pathophysiological phenomena, such as respiratory stimulation, pulmonary hypertension, arrhythmia, aldosterone secretion, cancers, anesthesia, neurological disorders, glucose homeostasis, and visual sensitivity. Therefore, they are important targets for innovative drug development. In this review, we emphasized the recent advances in our understanding of the biophysical properties, gating profiles, and biological roles of TASK channels. Given the different localization ranges and biologically relevant functions of TASK-1 and TASK-3 channels, the development of compounds that selectively target TASK-1 and TASK-3 channels is also summarized based on data reported in the literature.

## 1. Introduction

Ion channels play essential roles in a variety of cellular functions and are involved in the pathophysiology of a wide range of diseases. Currently, mammalian potassium channels are the most numerous, broadly distributed, and complicated class of ion channels that have been identified. They are classified into three main categories based on the topology of the transmembrane regions: voltage-gated potassium (K_v_) channels, inwardly rectifying potassium (K_ir_) channels, and two-pore domain potassium (K_2P_) channels [1,2,3]. Among them, the family containing K_v_ channels is the largest. It includes approximately 40 genes, which are divided into 12 subfamilies (K_v1_–K_v12_), and the channels mainly consist of six transmembrane fragments and a pore structural domain [4]. Furthermore, the calcium-activated potassium channels (K_Ca_) have either seven or six transmembrane segments and can link K^+^ dynamics to intracellular calcium signaling [5]. K_ir_ channels are defined as channels that permit the inward flow of K^+^ under hyperpolarization but permit little or no outward K^+^ flow under depolarization. They can be grouped into seven subfamilies (K_ir1–7_), and these channels have two transmembrane fragments and a pore structural domain [6]. K_2P_ channels have a dimeric structure instead of a tetrameric structure, and each subunit consists of four transmembrane fragments and two pore domains. These subunits are organized into six subgroups based on their various architectures and functions [7]. K_2P_ channels can be regulated by diverse chemical, physical, and metabolic factors, such as extra- or intra-cellular pH, temperature, mechanical stimuli, G proteins, and signaling lipids [8]. They play an important role in stabilizing resting membrane conductance and repolarizing the action potential of excitable cells [9]. 

The tandem of pore domains in a weakly inward rectifying K^+^ channel 1 (TWIK-1) was the first K_2P_ channel to be discovered and identified [10], and TWIK-related acid-sensitive K^+^ channel 1 (TASK-1) was the first cloned mammalian potassium channel that was found to create the characteristic background potassium currents [11]. TASK channels comprise TASK-1 (K_2P_3.1 or KCNK3), TASK-3 (K_2P_9.1 or KCNK9), and TASK-5 (K_2P_15.1 or KCNK15), which are composed of 394, 374, and 330 amino acids, respectively, in humans [11,12,13] (Table 1). Among them, TASK-1 shares 58.9% amino acid sequence homology with TASK-3 and 51.4% homology with TASK-5, whereas TASK-3 shares 55.1% homology with TASK-5 [14,15,16], and TASK-5 was not found to be functionally expressed in the recombinant system [13]. TASK channels are important members of the K_2P_ channel family and are widely involved in pathophysiological conditions. Therefore, an improved understanding of the biological mechanisms and functions of TASK channels will greatly assist in the future development of novel, potent, and selective target compounds.

In this review, we emphasize the recent advances in our understanding of the biophysical properties, gating profiles, and biological roles of TASK channels. Given the different localization ranges and biologically relevant functions of TASK-1 and TASK-3 channels, the development of compounds that selectively target either TASK-1 or TASK-3 channels is also summarized. 

## 2. The Structure, Localization, and Electrophysiological Properties of TASK Channels 

### 2.1. Structure

**Figure 1:**
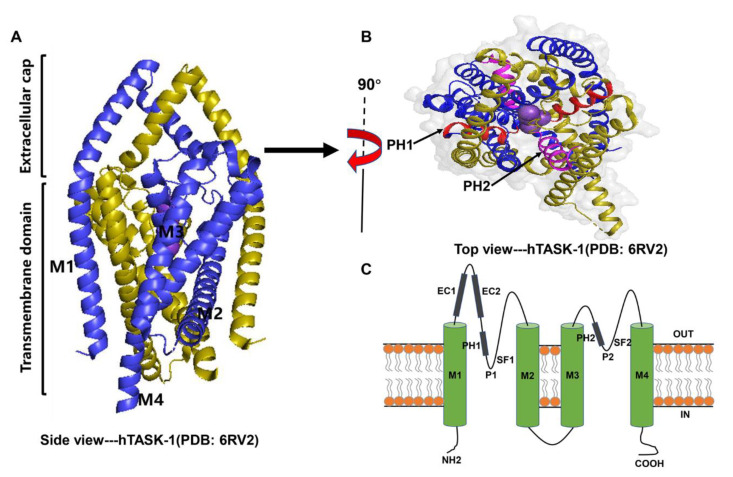
The structure and topology of the hTASK-1 channel. (**A**) Side view from the cell membrane. The hTASK-1 channel (PDB code 6RV2) consists of two homologous subunits, which are shown in blue and olive. The four transmembrane fragments (M1–M4) and two extracellular cap regions are labeled. (**B**) Top view of the hTASK-1 channel from the extracellular face, with the two pore helices (PH1 and PH2) highlighted in red and magenta in each subunit, respectively. The purple spheres are potassium ions. (**C**) Schematic topology of TASK channels: EC1 and EC2 are extracellular caps 1 and 2; P1 and P2 are pore regions 1 and 2; SF1 and SF2 are the selectivity filter of P1 and P2; OUT and IN represent the extracellular and intracellular surface, respectively.

As the closest homologue, the crystal structure of the human TASK-1 (hTASK-1) channel has currently been resolved at a resolution of 3.0 Å using X-ray crystallography [101]. Similar to other members of the K_2P_ family, TASK channels have two-fold symmetry, with each subunit containing four transmembrane α-helices (M1–M4), an inner pore region located between M1 and M2, an inner pore region located between M3 and M4, two pore helices (PH1 and PH2) suspended like cradles in their respective pores, and two selectivity filters (SF1 and SF2) shaped by four pore loops that interact with each other to determine the ionic selectivity of the channels [102] (Figure 1). The structures of TASK-1 channel are from protein data bank (PDB) and are generated by PyMOL 2.5. 

The special structure of the extracellular caps (EC1 and EC2) formed by the conserved M1-P1 linkers is a structural hallmark of all K_2P_ channels. These caps project externally and create two tunnel-like side gates in the membrane to allow for the bilateral exit of potassium ions [101,103]. The extracellular ion pathway (EIP) behind the selective filters also plays an important role in regulating the sensitivity of TASK channels to extracellular pH levels [104]. Most K_2P_ family members have conserved cysteines at the top of the cap structure that form a covalent disulfide bond between the two subunits and stabilize the dimer structure. Along with the tandem-pore domain halothane-inhibited K^+^ channels (THIK), TASK channels do not have cysteines at these positions, and in this case, the dimer is mainly stabilized by interactions between the hydrophobic residues at the cap domains [101,105,106] (Figure 1A,C). The cap domains are partly responsible for the insensitivity of the TASK-3 channel to external tetraethylammonium [107]. In addition, the M2 transmembrane helix of TASK channels bends near the center of the membrane, producing two “lateral fenestrations” that connect the pore region to the plasma membrane [108,109]. 

### 2.2. Expression and Localization 

The TASK-1 channel is abundantly expressed in the human central nervous system (CNS) and in peripheral tissues. In the human CNS, the highest expression levels of TASK-1 are in the cerebellum, thalamus, and pituitary gland, whereas the lowest expression level is in the corpus callosum. In human peripheral tissues, TASK-1 expression level is the highest in the pancreas, placenta, lungs and pulmonary arteries [17,18]. It is lower in the prostate, stomach, small intestine, and heart and lowest in the liver, spleen, skeletal muscles, and testes [17]. In rats and mice, the highest levels of TASK-1 mRNA in the CNS were observed in the cerebellum and somatic motoneurons, while the lowest levels were observed in the septum and striatum [16,110]. 

In humans, the TASK-3 channel is predominantly found in the CNS and is expressed in almost all regions of the brain, with the highest expression in the cerebellum and higher expression in the cerebral cortex, thalamus, nucleus accumbens, hippocampus, and hypothalamus. Its expression is lowest in the spinal cord, caudate nucleus, and corpus callosum. In addition, small amounts are expressed in peripheral tissues, such as the stomach, testes, skeletal muscles, uterus, kidney, spleen, pancreas, prostate, and small intestine. Very low levels are expressed in the heart, liver, and lungs [17]. In rat tissues, TASK-3 mRNA is widely expressed in the brain (with the highest expression levels found in the cerebellum), kidney, liver, lungs, colon, stomach, spleen, testes, and skeletal muscles. Its expression level is lowest in the heart and small intestine [71,110,111]. In humans, the TASK-5 channel is highly expressed in the pancreas and is also relatively abundantly expressed in the liver, kidney, lungs, ovary, testes, and heart [13].

### 2.3. Electrophysiological Properties

K_2P_ channels are active across the entire physiological membrane potential range and generate background leaked potassium currents [112]. TASK channels are important members of the K_2P_ channel family and have a current–voltage (I–V) relationship curve similar to the Goldman–Hodgkin–Katz equation at extra- and intra-cellularly different K^+^ concentrations, indicating that the generation of TASK channel currents correlates with K^+^ concentrations on both sides of the cell membrane. This also suggests that these channels show a high level of voltage-dependent gating despite the absence of a traditional voltage-activating domain because they have an ionic check valve located at the SF [113]. TASK channel currents are outwardly rectified and insensitive to potassium channel blockers, such as Ba^2+^, Cs^+^, TEA, and 4-AP [114]. TASK-1 and TASK-3 have different sensitivities to ruthenium red (RR or RuR), zinc, and anandamide. For instance, RR and zinc selectively block the TASK-3 channel but not the TASK-1 channel [115,116], whereas anandamide has a more potent blocking effect on TASK-1 than the TASK-3 channel [117]. In addition, TASK-1 and TASK-3 channel proteins can form functional heterodimers and are insensitive to mechanical forces [71,118]. The second messenger diacylglycerol can also inhibit TASK channels by activating G protein-coupled receptors [119,120].

**Figure 2:**
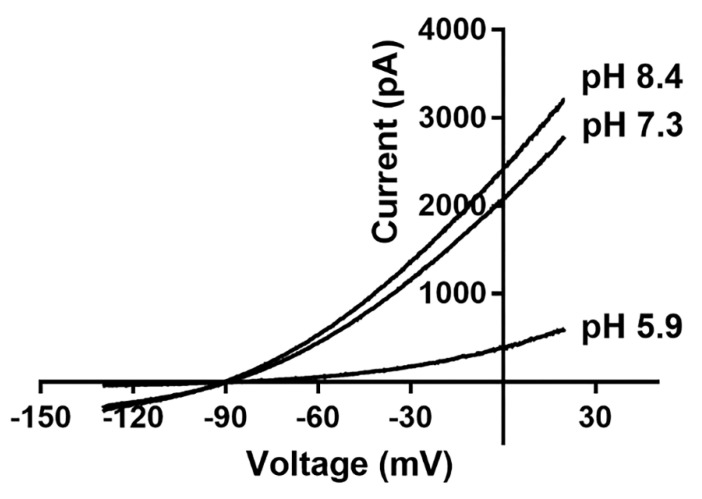
Representative illustration of the current−voltage (I−V) relationship for TASK channels under different extracellular pH conditions. TASK channels are sensitive to changes in extracellular pH; they are significantly inhibited by acids and are weakly activated by alkalis.

TASK channels are sensitive to changes in extracellular pH but not intracellular pH, and can be blocked by external protons [121]. The histidine residue at position 98, which is near SF1, and the aspartic acid residue at position 204, which is near SF2, are the key sites for sensing extracellular pH variations [104,122]. Extracellular membrane acidification significantly inhibits TASK channels, whereas alkalization weakly activates them (Figure 2). Variations in extracellular pH also affect the ion selectivity of TASK channels, with TASK-1, TASK-3, and TWIK-1 channels becoming less permeable to potassium ions and more permeable to sodium ions when the extracellular solution becomes acidified [123,124,125]. 

## 3. Gating Profiles

Gating mechanisms are defined as changes in the spatial conformation of ion channels that affect ion influx (the open state) or efflux (the closed state) [102,126]. Potassium ion channels can be classified depending on whether they use classical gating or “C-type” gating. Ion channels belonging to the classical gating class have separate gating and filter regions that function as channel switches and ion-selective filters, respectively. However, ion channels that use “C-type” gating do not have a single gating region. Instead, the gating mechanism is based on the SF, which performs two functions simultaneously [127]. Advances in resolving the 3D spatial structures of K_2P_ channel proteins, along with the discovery of a large number of small-molecule compounds capable of binding to them, have shown that there are multiple mechanisms for regulating their activity. However, the “N-type” inactivation mechanism, known as the “ball and chain” mechanism (generally rapid, with intracellular N-terminal amino acids obstructing the channel’s inner mouth after channel opening), has not been reported in K_2P_ channels [1], and the principal mechanism is “C-type” gating [128,129,130]. It is dependent on potassium-dependent order-disorder transitions in the SF region, with potassium ions as the voltage-sensing particles. The SF region also has adjacent loops that respond to gating cues relayed through the SF2-M4 loop [113,131]. For two-pore domain TASK potassium channels, there are currently three known main gating site mechanisms [132].

The first gating mechanism occurs at the vestibule site. Mutations and computational calculations of a number of different TASK channel antagonists, such as A293, A1899, PK-THPP, doxapram, ML365, and DR16.1, have shown that these antagonists interact with hydrophobic residues below the SF. The residues are located in the central cavity, and the previously listed antagonists inhibit channel activity by directly plugging the channel and preventing the flow of potassium ions [133,134,135,136]. These binding sites below the SF represent conserved sites of action, and studying them has resulted in a better understanding of the TASK channel gating profiles. It has been reported that the interactions of two crossed C-terminal M4 transmembrane helices at the intracellular vestibule entrance can form the so-called “X-gate”, which includes the six amino acids ^243^VLRFMT^248^ in the TASK-1 channel and ^243^VLRFLT^248^ in the TASK-3 channel [101]. Mutations within the “X-gate” and its surrounding regions markedly influence channel activity and its responses to the activities of volatile anesthetics, neurotransmitters, and G protein-coupled receptors [62,63]. In one study, Rödström et al. [101] identified two compounds, BAY1000493 and BAY2341237, that inhibit the TASK-1 channel. Both are potent, with IC_50_ values of 9.5 and 7.6 nM, respectively. In addition, both were found to bind within the inner vestibule region directly below the SF with low washout rates. This type of intracellular gating mechanism has not yet been found in other members of K_2P_ channels, with the exception of the alkaline-activated TASK-2 channel [137]. These studies will be beneficial for the future development and optimization of regulators that target TASK channels. 

The second gating mechanism occurs at the keystone inhibitor site. A cationic dye, trinuclear oxo-bridged ruthenium amine ruthenium red (RuR), can selectively inhibit the homodimeric TASK-3 channel by tethering two of its subunits, and the residue at position E70 is critical for this blocking activity [138]. Using a combination of mutagenesis, X-ray crystallographic, and electrophysiological studies of an RuR-insensitive TWIK-related K^+^ channel 1 (TREK-1), Pope et al. [139] showed that RuR acts by binding to an acidic residue pair that comprises the keystone inhibitor site, which is located under the extracellular cap domain archway directly above the SF. One ruthenium amine moiety in the RuR molecule binds directly over the channel pore at the S0 ion site, and the remainder binds to one of the two branches of the EIP [139,140]. Thus, the keystone inhibitor site creates an electrostatic and physical barrier that prevents the flow of potassium ions through the SF and EIP. They also showed that this “finger in the dam” mechanism is independent of “C-type” gate activation.

The third gating mechanism occurs at the “lateral fenestrations” site. Based on the most recently determined structure, there are three moving parts in K_2P_ channels. Of these, the M4 transmembrane helix is the most obvious [131,141,142,143]. Brohawn et al. [142,144] suggested that K_2P_ channels have two states: namely “up” and “down” states. The “up” state is primarily achieved by the lateral movement of the M4 helix at its midpoint, resulting in closure of the “lateral fenestrations.” In the “down” state, the M4 helix is nearly straight, crosses the membrane at an angle of approximately 45°, and make no contact with the M2 segment of the other subunit, leaving a 5–10 Å gap that is closest to the lipid bilayer. Molecular dynamics simulations of ion channels have revealed that the “lateral fenestration” site can bind to regulators and represents a target site that should be considered in pharmaceutical development [109]. Rinné et al. [145] proposed that the local anesthetic bupivacaine binds at the “lateral fenestration” sites of TASK channels, where it acts allosterically to disrupt K^+^-flux gating at the SF [113].

## 4. The Biological Roles of TASK Channels

Many physiological and pathological conditions are related to TASK channels, and they have a diverse distribution throughout the body. This suggests that the pharmacological regulation of TASK channel activity may be of great therapeutic value (Table 1). 

### 4.1. Breathing Rhythm

TASK channels are sensitive to extracellular pH and are expressed in multiple chemosensory nuclei or carotid bodies, which can sense PCO_2_ shifts in the early stages of acidosis [19,20,21]. The respiratory stimulant doxapram inhibits the activities of human TASK-1 and TASK-3 channels with effective half doses of 4 and 2.5 µM, respectively, suggesting that TASK channels are involved in regulating breathing rhythm [22]. Pharmacological and single-channel experiments revealed that TASK-1, TASK-3, and TASK-1/TASK-3 heterodimers play critical roles in the chemosensation of the carotid body and are major sensors of hypoxia and metabolic acidosis [23]. In neural recordings from electrodes implanted at the carotid body/sinus from TASK-1^−/−^ mice, a significant reduction (49% and 68%, respectively) in chemoafferent cell firing induced by hypoxia (10% O_2_) and hypercapnia (3–6% CO_2_) was found along with a blunted ventilatory response to hypoxia. No changes in TASK-3^−/−^ mice were observed under the same conditions, suggesting that TASK-1 in particular plays a key role in peripheral chemosensing. However, the TASK-3 channel can mediate hypoxic depolarization of normal glomus cells [24]. Studies using TASK-1 channel knockout (KO) mice also showed that pH sensitivity in serotonergic raphe neurons was abolished but was maintained in the retrotrapezoid nucleus (RTN). Because the RTN is central to the normal ventilatory response to CO_2_, this indicates that TASK-1 channels are not involved in the regulation of central respiratory chemosensitivity [25]. Alkaline-sensitive TASK-2 channels are widely distributed in Phox2b-expressing neurons in the brainstem RTN, which are the principal central respiratory chemoreceptors. TASK-2 KO mice displayed a diminished ventilatory reaction to CO_2_/H^+^ in vivo, indicating that TASK-2 channels play a major regulatory role in central respiratory chemoreception [1,20,137,146]. 

### 4.2. Pulmonary Artery Hypertension

Hypoxic pulmonary vasoconstriction (HPV) is an autoregulatory mechanism of the pulmonary vessels in response to hypoxia, which can reduce the ratio of ventilation and blood flow in the hypoxic alveolar region to ensure its oxygen supply. Thus, it plays an overriding role in maintaining the local ventilation/blood flow ratio and a constant arterial partial pressure of oxygen. Potassium channels play an important role in pulmonary arterial smooth muscle cells (PASMCs), and reduced potassium channel activity increases their resistance to apoptosis, cell proliferation, and vascular constriction, leading to vascular remodeling [147,148,149]. Hypoxia inhibits certain potassium channel activities, leading to cell membrane depolarization, which activates voltage-dependent calcium channels. This triggers inward extracellular Ca^2+^ flow and the contraction of pulmonary vascular smooth muscle, which ultimately causes increased pulmonary vascular resistance and the initiation of HPV. There are five main potassium channels in PASMCs: voltage-gated potassium (K_v_) channels, Ca^2+^-activated potassium (K_Ca_) channels, ATP-sensitive potassium (K_ATP_) channels, inward rectifier potassium (K_ir_) channels, and K_2P_ channels [150,151]. Human PASMCs express only TASK-1 potassium channels [26]. 

Pulmonary artery hypertension (PAH) is a devastating and lethal disease in which the loss of the TASK-1 channel causes cell depolarization, which in turn increases the tension of PASMCs, leading to PAH [26,27]. Loss of function of KCNK3, the gene that edits the TASK-1 channel, is an important contributor to the pathogenesis of PAH [28]. Six heterozygous missense variants of the TASK-1 channel were first reported in 2013, and all of them resulted in a loss of function and were found to be the cause of both familial and idiopathic PAH in patients [29]. Several more mutations in the TASK-1 channel have been identified since then [30,31,32,33,149]. However, whether pharmacological inhibition of the TASK-1 channel can also contribute to the development of PAH remains unclear. Vitamin D deficiency is strongly associated with pulmonary hypertension. Restoring vitamin D levels improves endothelial function and increases TASK-like potassium currents, and this can alleviate PAH [34,35]. It has been shown that pharmacological activation of KCNK3 with ONO-RS-082 in vivo can alleviate the symptoms of pulmonary hypertension (PH) caused by monocrotaline in rats [36]. Therefore, restoring KCNK3 expression in the pulmonary vasculature through gene therapy may be a remedy for PAH. 

### 4.3. Cardiac Arrhythmia

TASK-1 potassium channels are atrial-specific, and protein blotting experiments have shown that the TASK-1 channel in the human heart is expressed at 14–16-fold higher levels in the atria than that in the ventricles [152,153,154]. The TASK-1 channel is known to be involved in the pathophysiology of cardiovascular diseases [37,38,39]. It has been found that TASK-1 KO mice have a significantly prolonged action potential duration [40] and that the use of the TASK-1 blocker A293 significantly prolonged the action potential duration in rat ventricular muscle. This suggests that TASK-1 channels are not only involved in maintaining the resting membrane potential of cardiac myocytes but also help in regulating the outward current during the plateau phase of the action potential [41]. Class III antiarrhythmic drugs prolong action potentials and inhibit atrial and ventricular arrhythmias. Among these drugs, amiodarone inhibits open and closed TASK-1 channels in a concentration-dependent manner, with an IC_50_ value of 0.4 μM in oocytes. This is a possible mechanism for the efficacy of these agents [42,43]. 

Atrial fibrillation (AF) is the most common persistent arrhythmia, with a prevalence rate of up to 4% and an increasing trend in the population. It is strongly associated with increased mortality and stroke rates [155]. Although the risk of stroke can be significantly reduced with anticoagulation drugs, there is still a significant need for medicines that can treat other AF-related symptoms. It has been found that TASK-1 mRNA and protein levels in atrial tissue are upregulated in patients with chronic AF, which can result in the shortening of action potentials in these patients [44,45]. In addition, it has been found that the experimental TASK-1 channel inhibitor A293 can control the rhythm of persistent AF in a porcine large animal model [46]. Furthermore, the breathing stimulant compound doxapram inhibits human TASK-1 and TASK-3 channels with equal potency and can be used in a novel pharmacological strategy for cardioversion of acute episodes of paroxysmal AF as well as the rhythm control of persistent AF [47]. Several other experiments have also shown that the targeted inhibition of TASK-1 channels can effectively treat AF, confirming that it is an antiarrhythmic target in general [48,49,50,51]. In addition to the regulation of vascular tone in pulmonary arteries, TASK-1 channels also play an important vasomotor role in systemic arteries under specific conditions or at certain stages of ontogenesis. It was reported that TASK-1 channels oppose vasocontraction of renal arteries under conditions of metabolic alkalosis [156]. Moreover, TASK-1 channels play an important anticontractile role in systemic circulation of newborn rats [157]. Thus, potential vasopressor actions of TASK-1 channel blockers should be considered in the recently suggested TASK-1 channel-targeted treatment of cardiac arrhythmia.

### 4.4. Aldosterone Secretion

It has been found that TASK^−/−^ channel KO mice exhibit non-tumorigenic primary hyperaldosteronism with overproduction of autonomous aldosterone that was neither suppressed by high dietary sodium intake nor corrected using the angiotensin II receptor 1 antagonist candesartan. These results suggested that TASK channels are possible therapeutic targets for primary hyperaldosteronism [52]. Furthermore, statistical analyses indicated that KCNK3 (TASK-1) variants are associated with hyperaldosteronism and hypertension [53]. Another study reported that deleting TASK-1 and TASK-3 channels in the zona glomerulosa of the adrenal tissue causes aldosterone-driven angiotensin II-independent hypertension [54] Several other studies also indicated that TASK channels participate in the modulation of aldosterone secretion [55,56,57]. 

### 4.5. Pain

Tissue acidification can cause acute and chronic pain, and TASK channels are very sensitive to extracellular acidification. For example, exposing the channel to an extracellular pH of 6.0 can reduce its current by 96% [71,158]. Acid inhibition of TASK channels may be an important mechanism for the sustained depolarization of cells due to tissue acidification. This suggests that they could also be potential targets for tissue acidification-induced nociceptive transmission [159,160,161,162]. Several studies have shown that TASK channels are closely associated with pain [58,59,72]. 

Marsh et al. [60] found that the mRNA levels of TASK channels in the spinal dorsal root ganglion (DRG) were correlated with spontaneous foot lifting after intradermal complete Freund’s adjuvant injections, indicating that TASK channels are associated with inflammation-induced pain. The activity of TASK channels is also altered in pathological models of nerve injury. The mRNA expression of TASK-3 and TWIK-1 channels was found to be downregulated in L4-L5 DRG ipsilateral to the neuropathic lesion one week after spared nerve injury surgery, whereas TASK-1 channel expression remained unchanged [73]. In addition, it has been reported that the TASK-3 channel co-locates with the transient receptor potential cation channel subfamily M member 8 (TRPM8) in sensory neurons. TRPM8 is a cold- and menthol-activated ion channel that participates in thermosensation [163]. Liao et al. [74] designed a peripherally acting selective agonist, CHET3, for the TASK-3 channel and used it to show that the TASK-3 channel is an intrinsic regulator of pain sensation. They proved that CHET3 could attenuate thermal hyperalgesia and mechanical allodynia in different rodent pain models. They also predicted that CHET3 would bind under the SF and close to the M2 and M4 regions, which would alter the gating activity of the channel by affecting the SF conformation. García et al. [61] found that spinal TASK-1 and TASK-3 channels are involved in the regulation of inflammatory and neuropathic pain and that intrathecal pretreatment with the activator terbinafine could reduce formalin-induced flinching and nociceptive hyperalgesia in rats with neuropathy.

### 4.6. Anesthetics

#### 4.6.1. Volatile Anesthetics

TASK channels are expressed in CNS sites relevant to anesthetic actions and are the targets of many general anesthetics. Volatile anesthetics were the first group of TASK channel modulators to be extensively studied. Volatile anesthetics, such as halothane and isoflurane, activate TASK channels at clinically relevant concentrations to hyperpolarize cell membranes and reduce excitability [62]. This is especially true for brainstem motor and thalamocortical neurons and helps explain the loss of consciousness and motor ability that general anesthetics induce [164,165] It has been reported that the hyperpolarizing effects of halothane and isoflurane were reduced in TASK-1/TASK-3 KO mice, and the corresponding anesthetic effects of sedation, hypnosis, and immobilization were also diminished [64]. However, it has also been reported that high concentrations of isoflurane (0.8 mM) activate the TASK-3 channel and the heterodimeric TASK-1/TASK-3 channel, but inhibit the TASK-1 channel. This suggested that different concentrations of volatile anesthetics can regulate the activities of TASK channels in both directions [65] Studies have shown the presence of a binding region for volatile anesthetics in the structures of TASK channels, which includes the residue M159 at the cytoplasmic terminus of the M3 segment [66] and, in particular, residues 243 to 248 at the beginning of the cytoplasmic C-terminus, a region also known as the halothane-responsive region [63,101]. In addition, it has been shown that halothane and isoflurane can inhibit calcium current in smooth muscle cells of the coronary arteries to directly dilate coronary arteries and increase coronary flow during anesthesia [166].

#### 4.6.2. Local Anesthetics

Local anesthetics inhibit TASK channels with low affinity, and inhibiting TASK channels could reduce seizures caused by toxic concentrations of local anesthetics [67,68]. Using functional mutagenesis screens, in silico drug docking, and molecular dynamics simulations, Rinné et al. [145] showed that the binding site of bupivacaine is located in the “lateral fenestration” sites in TASK channels and that bupivacaine preferentially interacts with the second pore helices independent of the extracellular potassium concentration. This allosteric block mechanism disrupting the K^+^-flux gating at the selectivity filter resulted in an inward-rectification and then a voltage-dependent inhibition under symmetrical potassium concentrations in TASK channels. This mechanism is different from those of pore blockers, such as A1899, A293, and doxapram [167].

### 4.7. Cancers

The TASK-3 channel is highly expressed in breast cancer, ovarian carcinoma, and melanoma cells, and it has been suggested that it promotes tumor growth and proliferation [75,76,77,78,79,80,81,82]. Thus far, only the TASK-3 channel in the K_2P_ family has been identified to be expressed in the inner mitochondrial membrane, and silencing it induces apoptosis of human melanoma cells [83,84]. In addition, a study using a short hairpin RNA (shRNA)-mediated knockdown of the TASK-3 channel confirmed that the TASK-3 channel is involved in migration and cell survival in gastric cancer. Thus, it represents a potential therapeutic target for gastric cancer treatment [85]. Moreover, the addition of Y4, a monoclonal antibody against the TASK-3 channel extracellular domain, to KCNK9-expressing carcinoma cells can inhibit tumor growth and metastasis [86]. These confirmed that the TASK-3 channel is a promising target for the treatment of malignancies that express KCNK9 [87].

### 4.8. Neurological Activities and/or Disorders

#### 4.8.1. Sleep

Synchronized burst firing and tonic action potential generation in the thalamocortical system of the brain mainly occur during sleep and waking states, respectively [168]. The switch between these two firing modes is critically modulated by the bidirectional regulation of TASK and TWIK-related K^+^ channels (TREK) in thalamic relay neurons. 

It has been found that the genetic KO of TASK-1 did not change sleep/wake times in mice [169]. One study analyzed the locomotor activity and circadian rhythms of TASK-3 KO mice and found that, when compared to wild-type litter controls, both had normal circadian rhythms. However, TASK-3 KO mice had significantly increased nocturnal activity (38%), suggesting that the TASK-3 channel plays an important role in the regulation of sleep [88]. Another study based on continuous electroencephalogram and electromyogram recordings of TASK-3 KO mice revealed that they had a slower transition from wakefulness to sleep and more fragmented sleep episodes and rapid eye movement (REM) theta wave (4–9 Hz) oscillations during sleep [89,90]. 

#### 4.8.2. Mental Retardation

Mutations of G236R or A237D in the TASK-3 channel can lead to maternally inherited Birk–Barel intellectual disability syndrome (BBIDS), characterized by varying degrees of mental retardation, congenital hypotonia, generalized or proximal weakness, and craniofacial deformities, including an elongated face, bilateral temporal stenosis, severe micrognathia, cleft palate, depressed upper lip, short pharynx, atypical ear shape, upward arching eyebrows, and long eyelids [91,92,93]. An additional gain-of-function mutation in the TASK-3 channel (A237T), the non-steroidal anti-inflammatory drug flufenamic acid, and terbinafine can reduce the decrease in channel currents caused by the G236R mutation. This combination may provide a strategy for the treatment of BBIDS [94,95]. Inhibition of histone deacetylation also rescued the phenotype of mental retardation syndrome in the Birk–Barel mouse model, as shown by the second-generation histone deacetylase inhibitor CI-994 [96]. 

#### 4.8.3. Depression 

Depression is a polygenic and highly complex psychiatric disorder that involves multiple environmental and somatic developmental factors. It is often difficult to treat, is extremely heterogenous, and is a significant burden on society. Current pharmacological treatments for depression can cause many unwanted side effects. Furthermore, they do not have any effect on many patients [170,171]. TASK and TREK-1 channels have been found to be associated with the mechanisms of several depressive disorders [90,97]. The gene encoding TASK-3, KCNK9, is located on chromosome 8q24, and TASK-3 KO mice display antidepressant effects, implying that TASK-3 is a therapeutic target for antidepressant action [90]. A marker of depression treatment is the inhibition of REM sleep, which was significantly reduced in wild-type animals when fluoxetine was administered but did not have the same effect in mice lacking TASK-3 channels. This confirms the potential of TASK-3 channels as targets for the treatment of depression [98,99].

### 4.9. Other Roles

Several studies have shown that the TASK-1 channels found in the pancreas participate in the regulation of glucose homeostasis [69,70]. Using the highly specific “RNAscope” method of in situ mRNA hybridization in combination with pharmacological antagonists and genetic deletion tools, Wen et al. [100] reported the first evidence that the TASK-3 channel is abundantly expressed in retinal ganglion cells and plays a critical role in visual processing in the retina.

## 5. Development of Selectively Targeted Compounds

**Figure 3:**
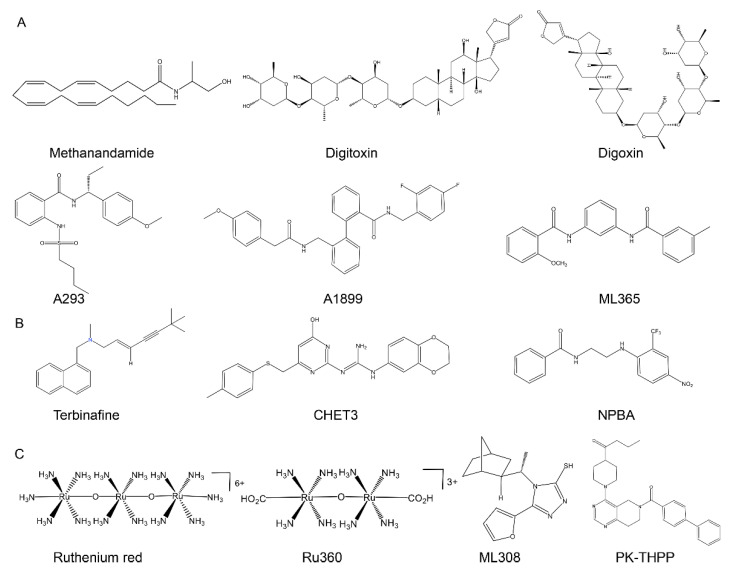
Chemical structures of compounds that target TASK-1 and TASK-3 channels. (**A**) The chemical structures of inhibitors targeting TASK-1 channel. (**B**) The chemical structures of activators targeting TASK-3 channel. (**C**) The chemical structures of inhibitors targeting TASK-3 channel.

TASK-1 and TASK-3 have different ranges of distribution and biologically relevant functions, even though they are closely related. Some compounds have been discovered that specifically target TASK-1 or TASK-3 channels, and using them has facilitated our understanding of the diverse molecular pharmacology and physiological roles of TASK channels (Table 2). The chemical structures can be seen in Figure 3.

### 5.1. Compounds That Target the TASK-1 Channel

Few compounds that specifically activate the TASK-1 channel have been discovered. Maingret et al. [117] found that methanandamide is a direct and relatively selective blocker of the TASK-1 channel, with an IC_50_ value of 0.7 μM. In addition, there is some evidence supporting the effect of anandamide on ion channels other than TASK-1, with effective concentration ranges for “side effect” and blockade of TASK-1 channels overlapping significantly. In the rat coronary artery, anandamide promoted the development of vasodilation by activating large-conductance Ca^2+^-activated K^+^ channels [178]. In isolated rat aortic smooth muscle cells, anandamide and methanandamide caused a decrease in the amplitude of the current mediated by K_v_ channels [179]. Thus, associating these effects with the direct effects of anandamide and methanandamide on potassium channels does not involve the activation of cannabinoid receptors. Cardiac glycosides have been used as antiarrhythmic drugs for many years, of which digitoxin and digoxin can selectively inhibit TASK-1 currents with IC_50_ values of 7.4 and 0.9 µM, respectively [172].

A-293 (AVE1231) is a TASK-1 selective inhibitor with IC_50_ values of about 0.22 against 0.95 μM of TASK-3 in *Xenopus* oocytes [41], and it can be used to treat atrial fibrillation [46,49]. In 2011, Decher et al. were the first to describe A-1899 (2″-[(4-methoxybenzoylamino)methyl]biphenyl-2-carboxylic acid 2,4-difluorobenzylamide) as a potent and highly selective small-molecule antagonist targeting the TASK-1 channel. It has IC_50_ values of 35.1 nM in *Xenopus* oocytes and 7 nM in CHO cells [173]. Bis-amide ML365 (2-methoxy-N-(3-(3-methylbenzamido)phenyl)benzamide) was also identified as a high-affinity blocker of the TASK-1 channel, with an IC_50_ value of 16 nM. It is a useful tool for in vitro studies of the biological functions and the development of pharmaceuticals that target the TASK-1 channel [135,174].

### 5.2. Compounds That Target the TASK-3 Channel

Wright et al. [95] showed that terbinafine, an antifungal agent, selectively activated TASK-3 currents with pEC_50_ of 6.2 (±0.12) by using the thallium flux assay, and no TASK-1 activity was observed during the electrophysiology studies. Liao et al. [74] developed and characterized the highly selective TASK-3 agonist CHET3 ((E)-1-(2,3-dihydrobenzo[b][1,4]dioxin-6-yl)-2-(4-hydroxy-6-((p-tolylthio)methyl)pyrimidin-2-yl) guanidine), which can selectively activate the TASK-3 channel with an EC_50_ of approximately 1.4 μM, and has no significant effect on TASK-1 channels, even at a concentration of 10 μM. Residues at the extracellular end of M2 (A105 and A108) and the intracellular end of M3 (E157) are important for TASK-3 channel activation, and these residues are not conserved in the TASK-1 channel. NPBA (N-(2-((4-nitro-2-(trifluoromethyl)phenyl)amino)ethyl)benzamide) binds to two distant clusters of residues and shows excellent potency and selectivity as a novel TASK-3 agonist, with an EC_50_ of 6.7 μM [175].

The trinuclear oxo-bridged RuR can selectively inhibit homodimeric TASK-3 but has no effect on either the TASK-1 homodimer and TASK-1/TASK-3 heterodimer, with an IC_50_ of 0.114 μM [138,139]. The dinuclear oxo-bridged ruthenium compound Ru360 can also selectively block the TASK-3 channel with an IC_50_ of 15.6 μM, which is at least 10-fold weaker than that of RuR because it carries half the positive charge of RuR [139]. Miller et al. [176] described that ML308 (4-[(1S)-1-[(1S,2R)-2-bicyclo [2.2.1]heptanyl]ethyl]-3-(furan-2-yl)-1H-1,2,4-triazole-5-thione) shows at least 50-fold selectivity for blocking the TASK-3 channel compared to the TASK-1 channel. It was identified as a novel selective inhibitor of the TASK-3 channel, with an IC_50_ value of 130 nM in a thallium influx fluorescent assay and 413 nM in an automated electrophysiology assay. PK-THPP (1-[1-[6-(4-phenylbenzoyl)-7,8-dihydro-5H-pyrido [4,3-d]pyrimidin-4-yl]piperidin-4-yl]butan-1-one) binds in the central cavity and is a relatively specific TASK-3 inhibitor. It has an IC_50_ value of 35 nM against TASK-3 but is approximately nine times weaker against TASK-1 channel, with an IC_50_ value of 303 nM [177]. 

## 6. Conclusions and Future Research Directions

Since the first identification of the human TASK-1 channel in 1997, we have learned a lot about the diversity and nature of the two-pore domain TASK potassium channels. In summary, recent advances in our understanding of TASK channels strongly suggest that they have enormous potential as a part of therapeutic strategies for multiple disorders. Given the diversity of K_2P_ channel binding sites and the possibility that there are over three gating sites for TASK channels, future research should focus on the discovery of other modulators, the further exploration of potential gating mechanisms, and on determining whether these binding sites can act in tandem. Furthermore, TASK-1 and TASK-3 channels share some biological roles. Therefore, it will be necessary to further clarify whether their relative contributions to a specific disorder differ in strength and duration, even though they are often co-expressed and can form heterodimers.

TASK-1 and TASK-3 channels have different distributions in the nervous system and peripheral tissues and are involved in the pathophysiological processes of a wide range of diseases. However, potent and specific compounds targeting the TASK-1 or TASK-3 channels have remained scientifically and economically underdeveloped. Relatively few small-molecule activators and inhibitors of therapeutic importance have been studied, and even fewer have entered clinical trials. Exploring the mechanism of action of TASK channels and using rational drug design to explore compounds that target the desired site of action should be high priorities for future research.

## Figures and Tables

**Table 1:** The classification and simple comparisons of mammalian TASK channels.

Channel	Gene Name	Length in Humans	Distribution in Humans	Indicative Biological Roles	References
TASK-1 (K_2P_3.1)	KCNK3	394	Abundantly expressed in the CNS and periphery. CNS: highest in the cerebellum, thalamus and pituitary gland, and lowest in the corpus callosum. Periphery: highest in the pancreas, placenta, lungs and pulmonary arteries, and lower in the prostate, stomach, small intestine, and heart, and lowest in the liver, spleen, skeletal muscle, and testis.	Chemosensation of breathing rhythm, pulmonary artery hypertension (PAH), cardiac arrhythmia (AF), aldosterone secretion, pain, general anesthesia, local anesthetic toxicity, glucose homeostasis	[11,14,15,16,17,18,19,20,21,22,23,24,25,26,27,28,29,30,31,32,33,34,35,36,37,38,39,40,41,42,43,44,45,46,47,48,49,50,51,52,53,54,55,56,57,58,59,60,61,62,63,64,65,66,67,68,69,70]
TASK-3 (K_2P_9.1)	KCNK9	374	Predominantly expressed in the CNS: highest in the cerebellum, higher in the cerebral cortex, thalamus, nucleus accumbens, hippocampus, and hypothalamus, and lowest in the spinal cord, caudate nucleus, and corpus callosum. Small amounts in the periphery: stomach, testis, skeletal muscles, uterus, kidneys, spleen, pancreas, prostate, and small intestine, and lowest in the heart, liver, and lungs.	Aldosterone secretion, pain, general anesthesia, local anesthetic toxicity, cancers, sleep, BBIDS, depression, visual sensitivity	[12,14,15,17,52,53,54,55,56,57,58,59,60,61,62,63,64,65,66,67,68,71,72,73,74,75,76,77,78,79,80,81,82,83,84,85,86,87,88,89,90,91,92,93,94,95,96,97,98,99,100]
TASK-5 (K_2P_15.1)	KCNK15	330	Highest in the pancreas, higher in the liver, kidneys, lungs, ovary, testis, and heart.	No reports	[13]

**Table 2:** List of selectively targeted compounds.

Channel	Activators	Compound Category	EC_50_ (μM)	Reference	Inhibitors	Compound Category	IC_50_ (μM)	Reference
TASK-1					Methanandamide	Cannabinoids	0.7 (MC)	[117]
					Digitoxin	Cardiac glycosides	7.4 (XO)	[172]
					Digoxin	Cardiac glycosides	0.9 (XO)	[172]
					A293	Small molecules	0.22 (XO)	[41]
					A1899	Small molecules	0.035 (XO), 0.007 (MC)	[173]
					ML365	Small molecules	0.016 (MC)	[135,174]
TASK-3	Terbinafine	Antifungals	NI	[95]	Ruthenium red	Cationic dyes	0.114 (XO)	[139]
	CHET3	Small molecules	1.4 (MC)	[74]	Ru360	Polynuclear ruthenium amines	15.6 (XO)	[139]
	NPBA	Small molecules	6.7 (MC)	[175]	ML308	Small molecules	0.413 (MC)	[176]
					PK-THPP	Small molecules	0.035 (MC)	[177]

XO: *Xenopus* oocytes, MC: mammalian cells, NI: not investigated.

## Data Availability

Not applicable.
